# Complete genome sequences of *Pseudomonas allii*, pathogens causing bacterial rot in onion, tomato, Chinese yam, and lettuce in Japan

**DOI:** 10.1128/mra.00113-25

**Published:** 2025-04-15

**Authors:** Yusuke Takashima, Ken Naito, Mamoru Satou, Hiroyuki Sawada

**Affiliations:** 1 Research Center of Genetic Resources, National Agriculture and Food Research Organization (NARO) 13516, Tsukuba, Ibaraki, Japan; The University of Arizona, Tucson, Arizona, USA

**Keywords:** *Pseudomonas*, plant pathogens

## Abstract

Here, we report the complete genome sequences of four strains of *Pseudomonas allii* (MAFF 301514, MAFF 302050, MAFF 730042, and MAFF 730146) that represent pathogens causing bacterial rot in onion, lettuce, Chinese yam, and tomato, respectively, in Japan.

## ANNOUNCEMENT

Bacteria-induced rot symptoms have been a challenge to onion cultivation in Japan, and *Pseudomonas marginalis* is considered as a causal agent (1). Previously, we re-identified *P. marginalis* strains obtained from onions as a novel species, *Pseudomonas allii* (2). During quality control in the Genebank Project of National Agriculture and Food Research Organization, three strains obtained from various crops (3–5, Table 1) showed nearly identical 16S rRNA and *rpoD* sequences to the type strain MAFF 301514. It is important to consider whether *P. allii* is a multi-host pathogen or an aggregate of heterogeneous strains, each with a limited host range (5). Here, we present the genome sequences of strains related to *P. allii*, including that of the type strain.

**TABLE 1:** Sequencing summary and statistics of the genome sequences of four strains of *Pseudomonas allii*

	Culture collection accession no.^*a*^			
Feature	MAFF 301514	MAFF 302050	MAFF 730042	MAFF 730146
Isolation sources	Onion (*Allium cepa*)	Lettuce (*Lactuca sativa* var. *capitata*)	Chinese yam (*Dioscorea* *polystachya* “Nagaimo”)	Tomato (*Solanum* *lycopersicum*)
Collection location	Hyogo	Tochigi	Aomori	Yamaguchi
Collection year	1980	1978	1977	1993
Original strain names	N-8042	NL 7843	YM 7702	To-1
Isolator	Akira Ohuchi	Yukio Tsuchiya	Manabu Umekawa	Tatsuhiko Karatsu
Sequencing summary				
Nanopore	DRR636248	DRR636249	DRR636250	DRR636251
No. of raw reads^*b*^/bases (Mbp)	23,920/306	35,212/379	39,707/576	22,714/341
*N*_50_ (bp)	24,261	18,716	25,540	26,573
Illumina	DRR636252	DRR636253	DRR636254	DRR636255
No. of raw reads/bases (Gbp)	3,744,092/0.56	12,388,708/1.85	14,074,772/2.10	10,552,470/1.57
Mean coverage of depth in polypolish	28×	94×	103×	80×
Genome statistics				
Total length (bp)	6,883,462	6,661,385	6,896,138	6,584,391
No. of circular replicons	1	1	1	1
Coverage of depth in Flye	32×	42×	62×	38×
G + C content (mol %)	60.9	60.9	60.8	61.0
No. of CDSs	6,238	6,015	6,274	5,939
Coding ratio (%)	89.7	89.8	89.6	89.8
No. of rRNA operons	5	5	5	5
No. of tRNA	71	73	73	72
CheckM^*c*^ (*Pseudomonas* marker set)				
Completeness (%)	100	100	100	100
Contamination (%)	0.72	0.72	0.72	0.72
OrthoANIu^*d*^ value (%)	–	99.1	98.9	99.1
dDDH^*e*^ value (%)	–	92.6	91.5	92.8
GenBank accession no.	AP039447	AP039448	AP039449	AP039450

^*a*^ Culture collection accession numbers (MAFF) under the Genebank Project of National Agriculture and Food Research Organization (https://www.gene.affrc.go.jp/databases-micro_search_en.php).

^*b*^ Signals were subjected to basecalling with a high-accuracy model (Guppy version 6.5.7, Oxford Nanopore Technologies [ONT]), demultiplexing, adapter trimming, and low-quality filtering with thresholds such as >1,000 bases and >7 Phred quality scores using MinKNOW version 23.04.5 (ONT).

^*c*^ CheckM (14) quality assessment implemented in DFAST was used.

^*d*^ OrthoANI using USEARCH. The values were calculated against the type strain (MAFF 301514) of *P. allii* using ANI Calculator on EzBioCloud. "–" indicates that the strain is used as a reference for comparison and no value can be shown.

^*e*^ dDDH, digital DNA-DNA hybridization. The values were calculated against the type strain (MAFF 301514) of *P. allii* using the Genome-To-Genome Distance Calculator version 3.0 (formula 2) with default options. "–" indicates that the strain is used as a reference for comparison and no value can be shown.

The strains were isolated from Honshu Island in Japan, including prefectures located at the northern (Aomori) and western (Yamaguchi) ends (Table 1), and were preserved under the Genebank Project as a lyophilized state. The strains were cultured on standard methods agar (Nissui) for 3 or 4 days at 28°C. Genomic DNA was extracted using the NucleoBond HMW DNA kit (Macherey-Nagel). Intact DNA was used for the library preparations.

A long-read sequencing library was prepared using the Rapid Barcoding Kit (SQK-RBK004, Oxford Nanopore Technologies [ONT]) and sequenced using PromethION with an R9.4.1 flow cell (FLO-PRO002, ONT). Raw ONT reads (Table 1) were filtered with >10 Phred quality score cutoff by Chopper version 0.5.0 (6). The filtered reads were assembled using Flye version 2.9.2 (7) with default options except for “-g 7 m --asm-coverage 50,” and the assembled circular genomes were polished using Medaka version 1.8.0 (https://github.com/nanoporetech/medaka) with the “r941_prom_hac_g507” model using the filtered reads.

Short-read sequencing libraries were prepared using Illumina DNA Prep (M) Tagmentation (Illumina), including the purification and size selection (ca. 500 bp), and 151 bp paired-end reads were sequenced using NovaSeq 6000 (Illumina) (Table 1). Adapter trimming and quality filtering were performed using fastp version 0.23.4 (8) with default settings except for the minimum length set to 151 and adapters specified by a sequencing company. Error corrections consisted of mapping with filtered Illumina reads using bwa-mem2 version 2.2.1 (9) with default except for “-a” option and polishing using Polypolish version 0.5.0 with default settings (10).

Genomes were annotated using the DDBJ Fast Annotation and Submission Tool (https://dfast.ddbj.nig.ac.jp/) with default settings except for the tRNAscan-SE and dnaA rotation function. Table 1 presents the statistics for the genomes determined in this study. OrthoANIu and digital DNA-DNA hybridization (dDDH) values (Table 1), calculated using the ANI Calculator (https://www.ezbiocloud.net/tools/ani) (11) and Genome-To-Genome Distance Calculator version 3.0 (https://ggdc.dsmz.de/ggdc.php#) (12), respectively, were higher than the Average Nucleotide Identity (ANI) (95%–96%) and dDDH (70%) thresholds for species delineation (13). These results show that the strains are the same species, and the genomes obtained here can be used as a starting point for analyzing intraspecific variations.

## Data Availability

Genome sequences and sequencing reads determined in this study were deposited in DDBJ/ENA/GenBank under Bioproject ID: PRJDB20186. Detailed accession numbers of genome sequences and sequencing reads were listed in Table 1.
